# Size‐Controlled Talc Nanosheet Ionogel Electrolytes for Dendrite Suppression in Solid‐State Sodium Metal Batteries

**DOI:** 10.1002/smsc.202500399

**Published:** 2025-08-31

**Authors:** Yuxing Gu, Yair Ein‐Eli, Woo Jin Hyun

**Affiliations:** ^1^ Department of Materials Science and Engineering Guangdong Technion – Israel Institute of Technology Shantou Guangdong 515063 P. R. China; ^2^ Department of Materials Science and Engineering Technion – Israel Institute of Technology Haifa 3200003 Israel; ^3^ Grand Technion Energy Program (GTEP) Technion – Israel Institute of Technology Haifa 3200003 Israel; ^4^ Israel National Institute of Energy Storage (INIES) Technion – Israel Institute of Technology Haifa 3200003 Israel; ^5^ Guangdong Provincial Key Laboratory of Materials and Technology for Energy Conversion Guangdong Technion – Israel Institute of Technology Shantou Guangdong 515063 P. R. China

**Keywords:** dendrite suppression, ionogels, sodium metal batteries, solid‐state electrolytes, talc

## Abstract

Ionogels composed of solid matrices and ionic liquids are promising candidates as solid‐state electrolytes for sodium (Na) metal batteries due to their nonflammability, high thermal stability, and desirable electrochemical and interfacial properties. Among various solid matrices, nanoscale materials are particularly attractive for increasing the mechanical modulus of ionogel electrolytes, contributing to the suppression of Na dendrite growth on metal anodes. However, the mechanistic understanding of this suppression remains limited. Here, size‐controlled talc nanosheets are introduced as solid matrices to investigate their influence on the ionogel modulus and the corresponding Na dendrite growth behavior, facilitating the rational design of ionogel electrolytes for dendrite suppression. Talc nanosheets with reduced lateral dimensions and thicknesses provide larger surface areas, enhancing the ionogel modulus through stronger immobilization of ionic liquids. High‐modulus ionogels with smaller nanosheets promote uniform Na deposition and reinforce the resistance of the electrolytes to vertical Na dendrite growth. Moreover, smaller talc nanosheets improve the Na‐ion transference number of ionogel electrolytes. The resulting talc nanosheet ionogel electrolytes enable Na_3_V_2_(PO_4_)_3_|Na cells to exhibit favorable rate capability at room temperature with capacity retention over 99% after 500 cycles at a rate of 0.5 C.

## Graphical abstract

Ionogel electrolytes are formulated using size‐controlled talc nanosheets for sodium metal batteries. Smaller nanosheets enhance ionic liquid immobilization and increase ionogel modulus. These high‐modulus ionogels improve Na deposition uniformity and mechanical resistance to dendrite growth. The resulting electrolytes enable the fabrication of solid‐state Na_3_V_2_(PO_4_)_3_|Na batteries with favorable rate capability and exceptional cycling stability at room temperature.

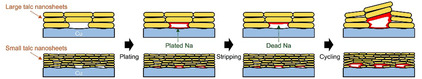

## 1 Introduction

Sodium (Na)‐based rechargeable batteries have attracted significant interest as a promising alternative to traditional lithium‐ion batteries due to the abundance of Na resources.^[^ ^1^, ^2^ ^]^ Among these, sodium metal batteries (SMBs) are particularly appealing because Na metal anodes offer a high theoretical capacity (1166 mAh g^−1^) and a low redox potential (−2.71 V vs. standard hydrogen electrode), leading to high energy density. However, the practical deployment of SMBs faces critical challenges. Conventional organic liquid electrolytes used in SMBs commonly undergo side reactions with the Na metal, resulting in uneven Na plating/stripping and serious dendrite growth, which can cause internal short circuits or battery failure.^[^ ^3^, ^4^, ^5^ ^]^ In addition, the flammability of organic liquid electrolytes raises safety concerns, including the risk of electrolyte leakage.^[^ ^6^, ^7^ ^]^ Consequently, considerable efforts have recently been directed toward developing solid‐state electrolytes for SMBs. Inorganic solid electrolytes exhibit excellent mechanical strength, preventing Na dendrite penetration, and offer nonflammability and high thermal stability for elevated‐temperature operation.^[^ ^8^, ^9^ ^]^ However, their surface roughness and rigidity often result in large interfacial resistance with electrodes.^[^ ^10^, ^11^ ^]^ Compared to inorganic solid electrolytes, polymer electrolytes are more mechanically flexible and easier to process, enabling better interfacial contact with electrodes and simplifying battery fabrication, but they suffer from low ionic conductivity at room temperature, limiting their electrochemical performance.^[^ ^12^, ^13^ ^]^ To address these limitations of inorganic and polymer electrolytes, gel electrolytes have been developed by incorporating liquid components into solid matrices.^[^ ^14^, ^15^, ^16^, ^17^ ^]^ While being confined by the solid matrices to form solid‐state electrolytes, the liquid components facilitate ion transport, leading to desirable room‐temperature ionic conductivity. Furthermore, the liquid phase improves contact between the solid‐state electrolytes and electrodes, reducing interfacial resistance.

Ionogels, a class of gel electrolytes, are composed of solid matrices and ionic liquids (ILs).^[^ ^18^, ^19^ ^]^ Unlike gel electrolytes that use common organic solvents at the cost of compromising safety,^[^ ^20^, ^21^ ^]^ ionogel electrolytes are formed with ILs that offer nonflammability, negligible vapor pressure, and high thermal stability, improving safety and extending the high‐temperature limit of battery operation. Moreover, strong immobilization of ILs within solid matrices provides mechanical integrity to maintain separation between the cathode and anode and imparts resistance against the dendrite growth of metal anodes. Among various solid matrices explored, nanoscale matrices have drawn particular interest due to their potential to increase the mechanical modulus and ion transport of ionogel electrolytes.^[^ ^22^, ^23^, ^24^, ^25^, ^26^ ^]^ While enhancing the mechanical modulus has been investigated with nanoscale solid matrices as a key strategy to strengthen the resistance of ionogel electrolytes to dendrite growth,^[^ ^27^, ^28^, ^29^ ^]^ the influence of nanoscale matrix size and ionogel modulus on Na dendrite growth remains largely unexplored. This limited understanding hinders the rational design of high‐modulus ionogel electrolytes for SMBs and impedes the elucidation of their dendrite suppression mechanism.

Here, we report high‐modulus ionogel electrolytes using size‐controlled talc nanosheets and Na‐based IL for dendrite suppression in SMBs. Talc nanosheets have been shown to suppress the formation of polynuclear complexes between charge carrier cations and IL anions in ionogel electrolytes, thereby enhancing the mobility of the charge carrier cations.^[^ ^24^ ^]^ However, the effect of nanosheet size on the mechanical modulus of ionogels has not been systematically investigated. Moreover, the relationship between mechanical modulus and dendrite growth behavior has received limited attention, despite its broader relevance beyond talc to other nanoscale solid matrices. In this study, talc nanosheets with varied lateral dimensions and thicknesses are prepared via solution‐based exfoliation of bulk talc microparticles and employed as solid matrices to formulate ionogels. Smaller nanosheets increase the ionogel modulus due to their larger surface areas, which enhance IL immobilization and promote more effective solidification. Na plating/stripping tests using Na|copper (Cu) cells reveal that ionogel electrolytes based on smaller nanosheets improve the uniformity of Na deposition on electrodes, and their higher modulus provides greater mechanical resistance to vertical Na dendrite growth, resulting in significantly extended cycling. Despite the increased modulus, the ionogel electrolytes exhibit Na‐ion conductivity comparable to that of the neat IL. Finally, Na_3_V_2_(PO_4_)_3_ (NVP)|Na cells assembled with the ionogel electrolytes based on the size‐controlled talc nanosheets demonstrate superlative rate capability and outstanding cycling stability at room temperature.

## 2 Results and Discussion

### 2.1 Size‐Controlled Talc Nanosheets

**Figure 1:**
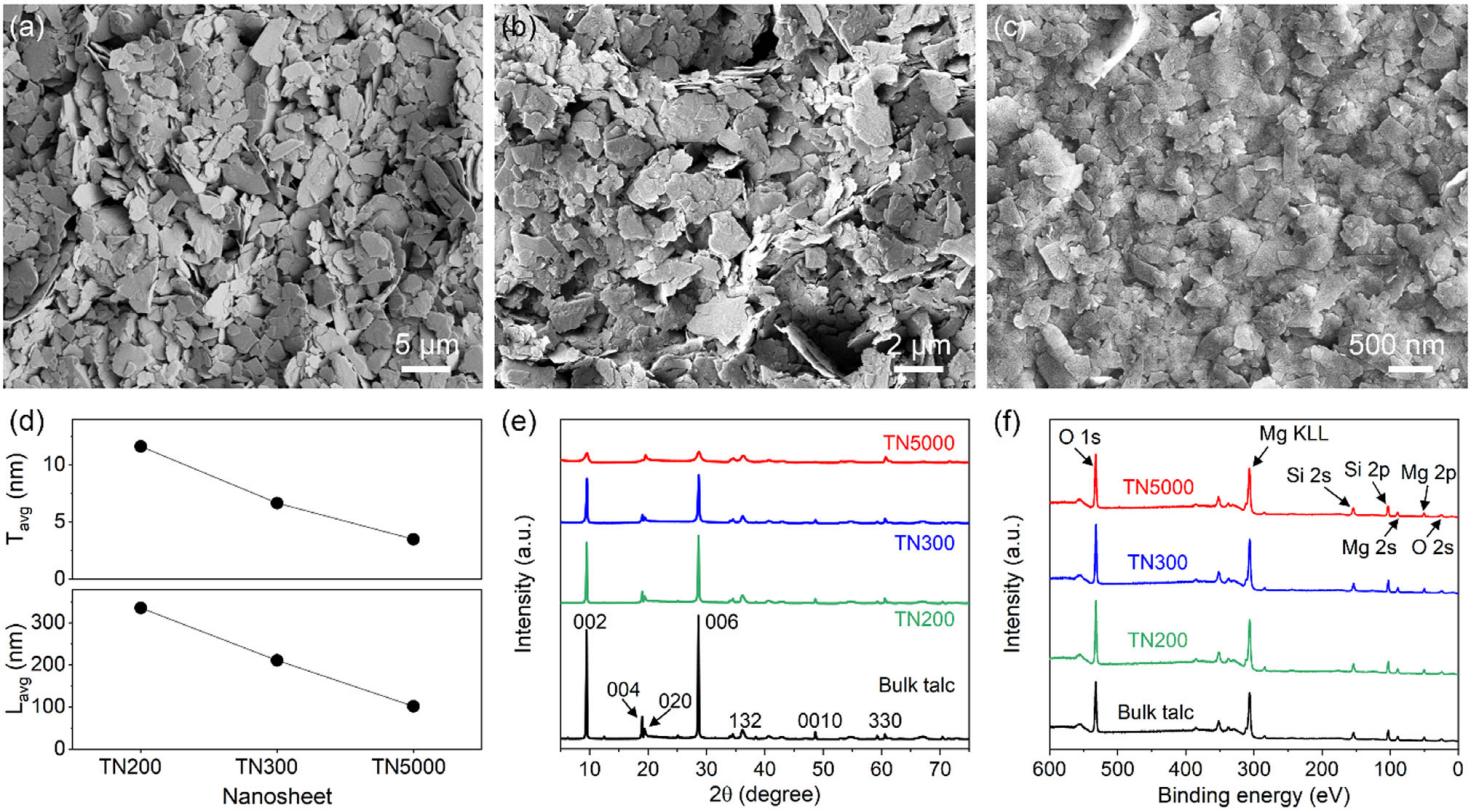
Size‐controlled talc nanosheets. a–c) SEM images of solution‐exfoliated talc nanosheets that were collected by centrifugation at different rotational speeds of 200 (a), 300 (b), and 5000 (c) rpm, referred to as TN200, TN300, and TN5000, respectively. d) Average thickness (*T*_avg_) and lateral size (*L*_avg_) of the nanosheets characterized by AFM. e,f) XRD patterns (e) and XPS spectra (f) of the talc nanosheets and bulk talc. The XRD peaks were indexed according to the ICDD database (no. 00‐019‐0770).

Talc nanosheets were prepared from bulk talc microparticles (Figure S1, Supporting Information) using a solution‐based exfoliation method (see Experimental Section for details). Talc consists of hydrous magnesium silicate layers held together by van der Waals forces, with each layer comprising an octahedral magnesium hydroxide plane sandwiched between two tetrahedral silicate planes. By disrupting these van der Waals forces in solution, bulk talc was exfoliated into nanosheets. To obtain nanosheets of different sizes, the exfoliated talc was collected by centrifugation at varied rotational speeds of 200 (**Figure** **1**a), 300 (Figure 1b), and 5000 (Figure 1c) rpm (Figure S2, Supporting Information), denoted as TN200, TN300, and TN5000, respectively. Higher rotational speeds generate greater centrifugal forces, allowing the collection of nanosheets with smaller thicknesses and lateral sizes. Atomic force microscopy (AFM) measurements (Figure S3, Supporting Information) showed that the average thickness and lateral size (Figure 1d and Figure S4, Supporting Information) were 11.8 and 350 nm, respectively, for TN200, 6.7 and 211 nm for TN300, and 3.5 and 98 nm for TN5000.

Figure 1e compares X‐ray diffraction (XRD) patterns of the bulk talc and exfoliated talc nanosheets. The exfoliated nanosheets retained the major XRD peaks of the bulk talc, implying that the crystalline structure was largely preserved. However, their (00*l*) peak intensities were reduced, indicating lower crystallinity along the vertical direction due to decreased thickness after exfoliation. Specifically, the relative peak intensity ratio of (004) to (020) (Figure S5, Supporting Information) decreased for talc nanosheets obtained at higher centrifugation speeds, reflecting their reduced thickness.^[^ ^30^, ^31^ ^]^ Moreover, as shown in Figure 1f, the exfoliated talc nanosheets of different sizes showed X‐ray photoelectron spectroscopy (XPS) spectra similar to that of the bulk talc, with distinct oxygen, magnesium, and silicon peaks and insignificant changes in atomic composition (Figure S6, Supporting Information). These XPS results reveal that, despite the decrease in thickness, the chemical structure of talc remained nearly unchanged after the solution‐based exfoliation, regardless of the centrifugation speed for the collection of the nanosheets.

### 2.2 Mechanical and Ion Transport Properties

**Figure 2:**
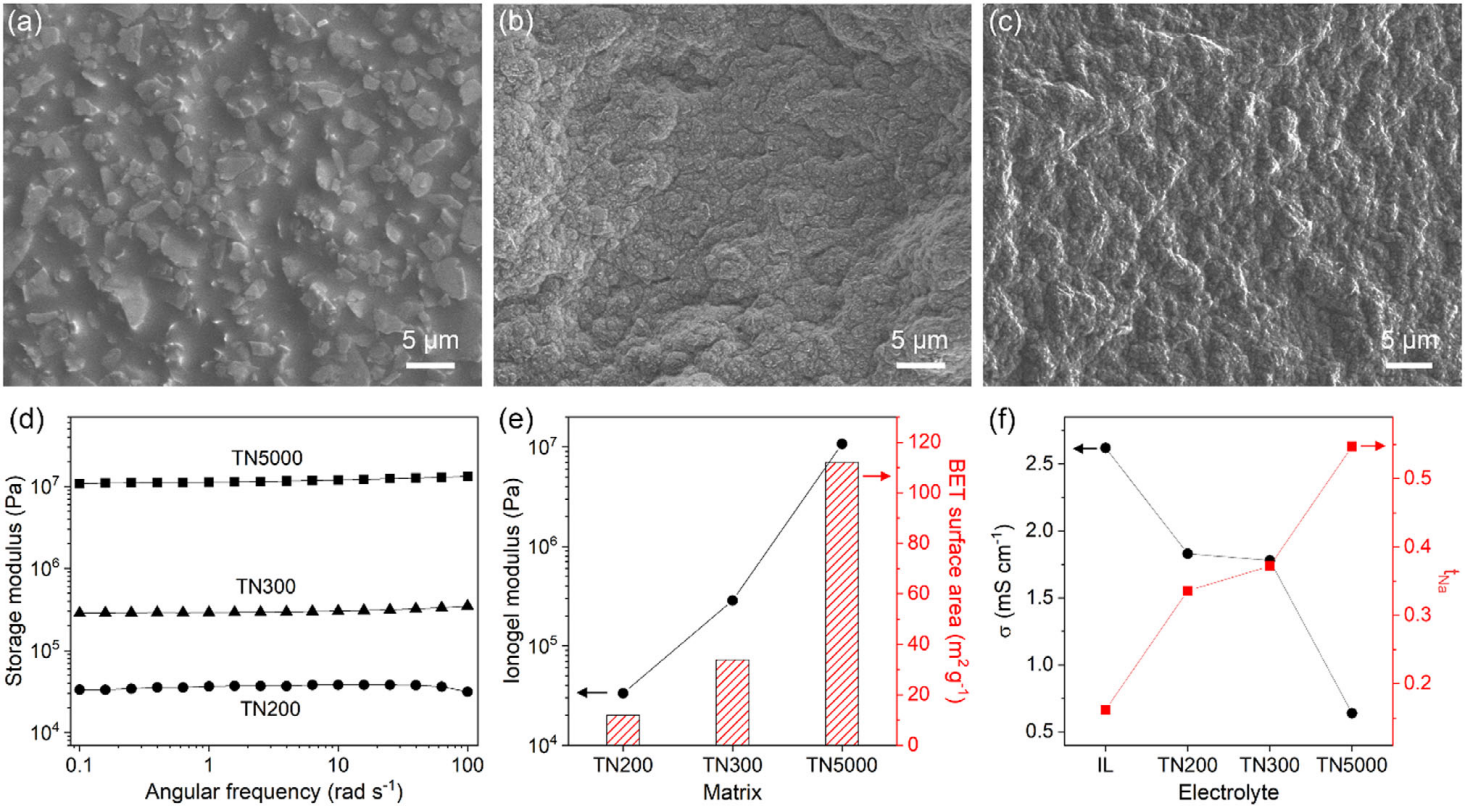
Mechanical and ion transport properties of talc nanosheet ionogels. a–c) SEM images of ionogels employing TN200 (a), TN300 (b), and TN5000 (c) as solid matrices. d) Storage modulus of the ionogels as a function of angular frequency. e) Ionogel modulus (storage modulus at 0.1 rad s^−1^) using TN200, TN300, and TN5000 matrices and BET surface area of the nanosheets. f) Ionic conductivity (*σ*) and sodium (Na)‐ion transference number (*t*_Na_) of the ionic liquid (IL) and ionogel electrolytes at room temperature.

Ionogels were formulated by mixing the exfoliated talc nanosheets with a Na‐based IL, *N*‐propyl‐*N*‐methylpyrrolidinium bis(fluorosulfonyl)imide (Py13FSI) containing 1 M Na bis(trifluoromethane)sulfonimide (NaTFSI) salt. In these ionogel electrolytes, the talc nanosheets interact with IL ions but do not serve as electrochemically active components for battery operation. Ionogels typically show a trade‐off between mechanical modulus and ionic conductivity depending on the solid matrix‐to‐IL ratio. A higher matrix content increases the modulus but reduces ionic conductivity.^[^ ^18^ ^]^ For all ionogels, the solid matrix‐to‐IL ratio was maintained at 2:3 to eliminate the effect of solid matrix loading on their properties. Scanning electron microscopy (SEM) showed that after mixing, TN200 (**Figure** **2**a), TN300 (Figure 2b), and TN5000 (Figure 2c) were uniformly coated with the IL, forming homogeneous ionogels.

To evaluate their mechanical strength, the storage modulus (Figure 2d) of the ionogels was measured. The storage modulus was higher than the loss modulus with minimal frequency dependence (Figure S7, Supporting Information), indicating reliable solid‐like behavior (Figure S8, Supporting Information), regardless of the nanosheet size. However, as the nanosheet size decreased, the mechanical strength (storage modulus) increased noticeably, which can be explained by the specific surface area of the nanosheets. These ionogels form through interactions between the IL and talc nanosheets, including electrostatic attraction and hydrogen bonding.^[^ ^24^ ^]^ Electrostatic attraction can occur between the positively charged IL cations and the negatively charged oxygen atoms on the basal surfaces of the silicate planes in talc, while hydrogen bonding can form between the oxygen atoms of the IL anions and the hydroxyl groups in the magnesium hydroxide layer of talc.^[^ ^24^ ^]^ These interactions contribute to the physical solidification of the ionogels, affecting the mechanical modulus and ionic conductivity. Smaller nanosheet sizes provide larger surface areas, promoting the interactions and thus leading to stronger IL immobilization, which strengthens the mechanical modulus of the ionogels. This explanation is supported by similar trends in the mechanical modulus and specific surface area with decreasing nanosheet size, as shown in Figure 2e. As a result, the TN5000 ionogel (1.1 × 10^7^ Pa at 0.1 rad s^−1^) exhibited an improved mechanical modulus by nearly three orders of magnitude compared to the TN200 ionogel (3.3 × 10^4^ Pa at 0.1 rad s^−1^). Furthermore, despite their high mechanical modulus, the deformable nature of ionogels allowed favorable interfacial compatibility (Figure S9, Supporting Information), facilitating conformal contact with electrodes for battery assembly.

The ion transport properties of the ionogel electrolytes were also investigated by characterizing their ionic conductivity (Figure S10, Supporting Information) and Na‐ion transference number (Figure S11, Supporting Information), as shown in Figure 2f. The ionic conductivity of the ionogels was lower than that of the IL alone, which can be ascribed to restricted ion‐conductive pathways due to the volume occupied by the solid matrices in the ionogels. Moreover, the reduced mobility of IL ions interacting with talc nanosheet surfaces contributed to the lower ionic conductivity. This contribution became more significant for smaller nanosheet sizes due to their large surface area, further reducing the ionic conductivity. In contrast to the ionic conductivity, the Na‐ion transference number of the ionogels was higher than that of the Na‐based IL, suggesting that the interactions between the IL and talc nanosheet surfaces disturb coordination between Na ions and anions and decrease the tendency of Na‐ion complex formation,^[^ ^24^ ^]^ improving Na‐ion mobility. This interpretation is supported by the observed increase in the Na‐ion transference number with the decrease in the nanosheet size. Consequently, despite their reduced ionic conductivity, the ionogels (0.4 mS cm^−1^ with TN5000) showed Na‐ion conductivity comparable to that of the IL (0.4 mS cm^−1^), as determined by the product of the ionic conductivity and the Na‐ion transference number.

### 2.3 Resistance to Na Dendrite Growth

**Figure 3:**
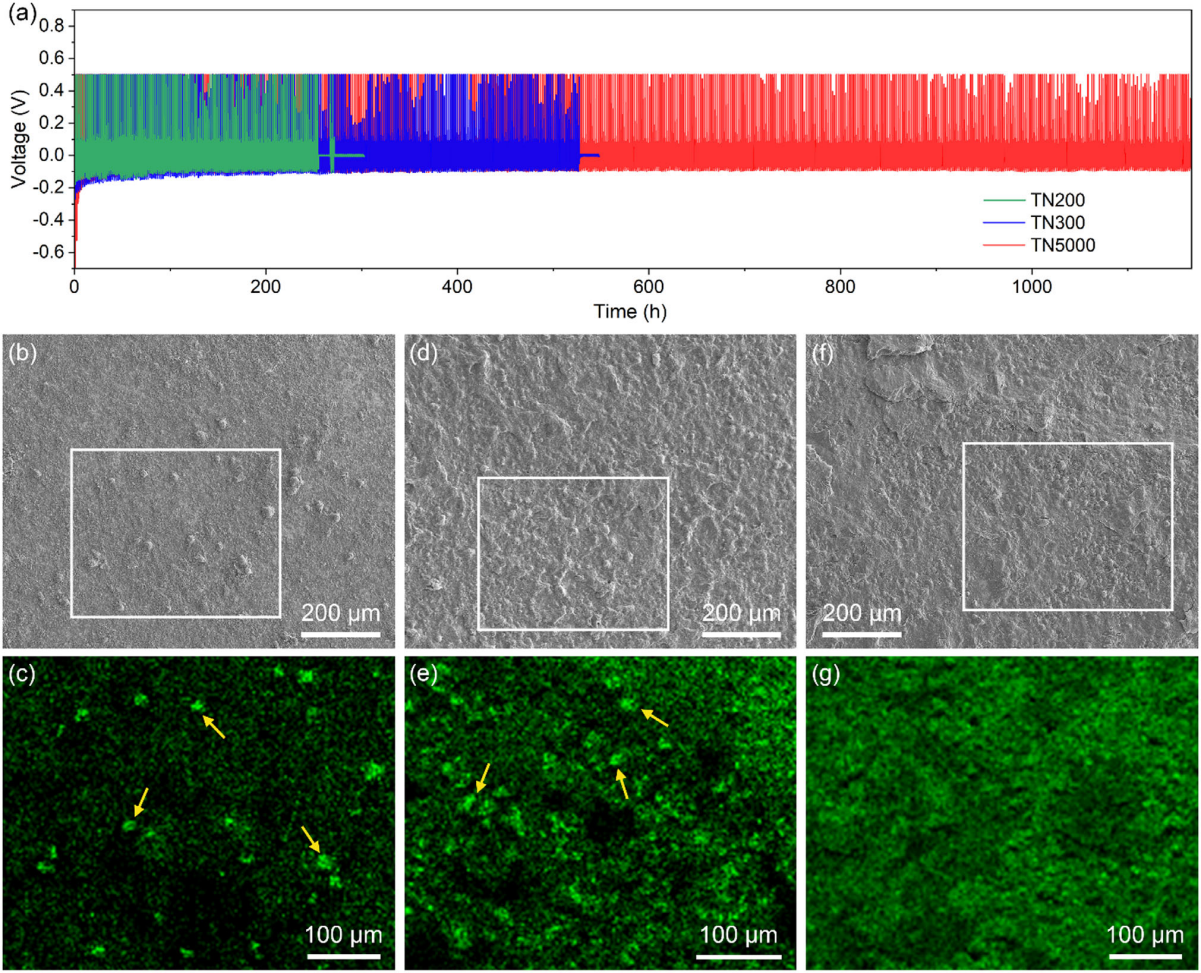
Na plating/stripping tests and EDS analysis. a) Voltage profiles of Na|Cu cells using TN200, TN300, and TN5000 ionogel electrolytes during plating/stripping cycles at a current density of 0.1 mA cm^−2^. Plating was performed for 1 h, and stripping was conducted with a cutoff voltage of 0.5 V. b,d,f) SEM images of Cu electrodes collected from Na|Cu cells using TN200 (b), TN300 (d), and TN5000 (f) ionogel electrolytes after the 10th plating step in cycling tests at a current density of 0.05 mA cm^−2^. c,e,g) EDS mapping images for the Na distribution on the Cu electrodes from the cells using TN200 (c), TN300 (e), and TN5000 (g) ionogel electrolytes. The mapping was performed on the regions marked by boxes in the corresponding SEM images.

Na|Cu cells were assembled with TN200, TN300, and TN5000 ionogel electrolytes to investigate the effects of nanosheet size and electrolyte modulus on Na dendrite growth during repeated plating/stripping cycles. **Figure** **3**a displays the voltage profiles of Na|Cu cells cycled at a current density of 0.1 mA cm^−2^, where Na was plated onto Cu electrodes for 1 h and stripped with a cutoff voltage of 0.5 V. The cycling stability varied significantly, depending on the ionogel electrolyte. The cell with the TN200 ionogel electrolyte experienced a short circuit after 131 cycles (Figure S12a, Supporting Information), whereas the TN300 ionogel electrolyte enabled extended cycling for 268 cycles (Figure S12b, Supporting Information). The TN5000 ionogel electrolyte further prolonged cycling to over 600 cycles (Figure S12c, Supporting Information), presenting substantially improved Na plating/stripping stability.

To better understand the enhanced cycling stability, Na|Cu cells using the ionogel electrolytes were disassembled after the 10th plating step during plating/stripping cycles at 0.05 mA cm^−2^, and Cu electrodes were collected and analyzed by energy‐dispersive spectroscopy (EDS), detecting deposited Na. The Cu electrode of the TN200 ionogel cell (Figure 3b,c) showed nonuniformly distributed Na islands with sizes of ≈10 μm (indicated by arrows in the EDS mapping image), implying that Na deposition was concentrated on localized points. This localized deposition on the Na islands can accelerate vertical Na growth, potentially leading to short circuits, as observed in the plating/stripping tests. Compared to the TN200 ionogel, the TN300 ionogel (Figure 3d,e) enabled less localized Na deposition on the Cu electrode, although Na islands of similar sizes were still observed. However, the Cu electrode of the TN5000 ionogel cell (Figure 3f,g) exhibited considerably improved Na deposition uniformity without noticeable island formation. These deposition patterns reveal that the ionogels with smaller nanosheet sizes and higher moduli promote more homogeneous Na plating, which explains the prolonged cycling stability observed in the plating/stripping tests.

**Figure 4:**
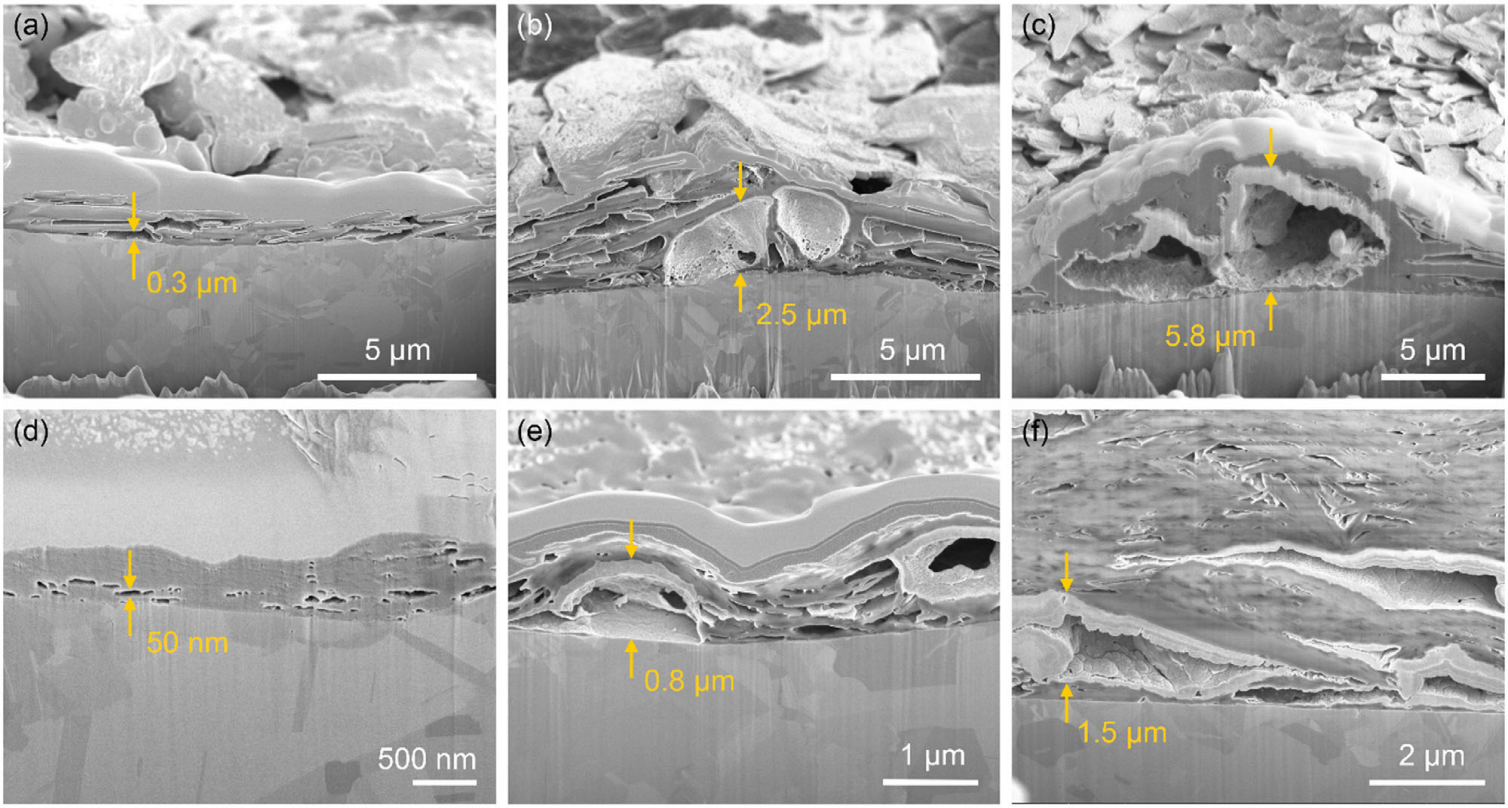
Growth of plated Na over cycling. a–c) Cross‐sectional SEM images of Cu electrodes from Na|Cu cells using the TN200 ionogel electrolyte after the 1st (a), 5th (b), and 10th (c) plating steps during plating/stripping cycles at a current density of 0.05 mA cm^−2^. Plating was conducted for 1 h, and stripping was executed with a cutoff voltage of 0.5 V. d–f) Cross‐sectional SEM images of Cu electrodes from Na|Cu cells employing the TN5000 ionogel electrolyte after the 1st (d), 5th (e), and 10th (f) plating steps.

To further elucidate the plating behavior, Na deposited between ionogels and Cu electrodes in Na|Cu cells using the TN200 and TN5000 ionogel electrolytes was examined by focused ion beam SEM (FIB‐SEM) after the 1st, 5th, and 10th plating during plating/stripping cycles at 0.05 mA cm^−2^, as shown in **Figure** **4**. After the 1st plating (Figure 4a), the TN200 ionogel cell showed voids at the ionogel/electrode interface, which were a few micrometers wide and several hundred nanometers high. Following the 5th plating (Figure 4b), plated Na became more apparent in some enlarged voids, where it was deposited along the inner surfaces. After the 10th plating, the voids containing plated Na expanded considerably (Figure 4c), which may correspond to the Na islands observed during EDS analysis. In contrast, the TN5000 ionogel cell exhibited significantly smaller voids after the 1st plating (Figure 4d). While these voids also expanded with repeated plating/stripping cycles (Figure 4e), observations after the 10th plating (Figure 4f) revealed that Na deposited within the voids grew more laterally than vertically. The voids appeared to be compressed downward, indicating constrained vertical Na growth under the higher‐modulus ionogel using TN5000.

**Figure 5:**
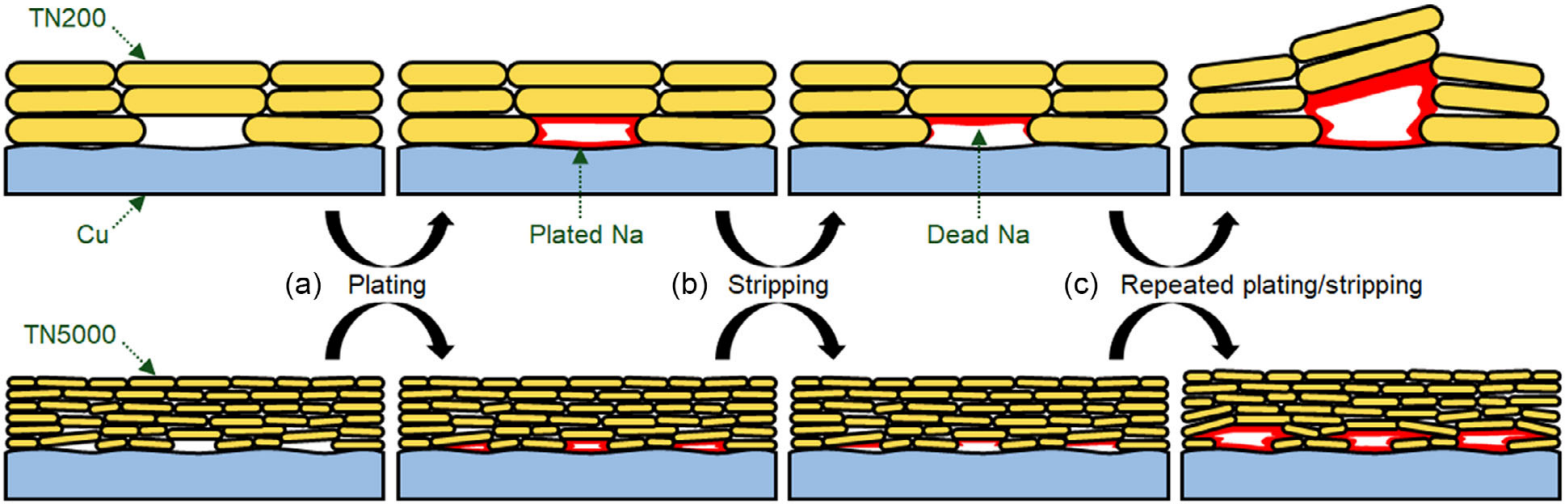
Schematic illustration of Na plating behavior during repeated Na plating/stripping on Cu electrodes using TN200 (top) and TN5000 (bottom) ionogel electrolytes. a) Na deposition concentrated in gaps formed by incomplete contact between talc nanosheets and Cu electrodes during plating. b) Creation of dead Na electrically isolated from the Cu electrodes during stripping. c) Expansion of the gaps due to the accumulation of dead Na and repeated Na plating during cycling.

**Figure** **5** compares the Na plating behavior with the TN200 and TN5000 ionogel electrolytes based on the EDS and FIB–SEM results. Irregular arrangements of talc nanosheets in the ionogels create gaps between the solid matrices and the electrode, which are filled with the IL. The COMSOL simulation (Figure S13, Supporting Information) reveals that the applied current during Na plating is concentrated in these gaps, making them preferential sites for Na nucleation (Figure 5a). During stripping (Figure 5b), Na preferentially dissolves near the base of the plated region,^[^ ^4^, ^32^ ^]^ where electron transfer initiates earlier, leaving behind electrically isolated Na (dead Na). With continued cycling (Figure 5c), the accumulation of dead Na and repeated Na plating generate localized mechanical stresses, gradually enlarging the gaps. Compared to the TN200 ionogel, the TN5000 ionogel employing smaller nanosheets produces smaller and more distributed gaps, reducing the localization of current concentration and nucleation sites. In addition, the higher modulus of the TN5000 ionogel exerts greater mechanical pressures against the Na growth force applied to the nanosheets, thereby enhancing the resistance to vertical Na growth. As a result, the TN5000 ionogel with smaller nanosheets facilitates more uniform and stable Na plating/stripping over repeated cycles.

### 2.4 SMB Performance

**Figure 6:**
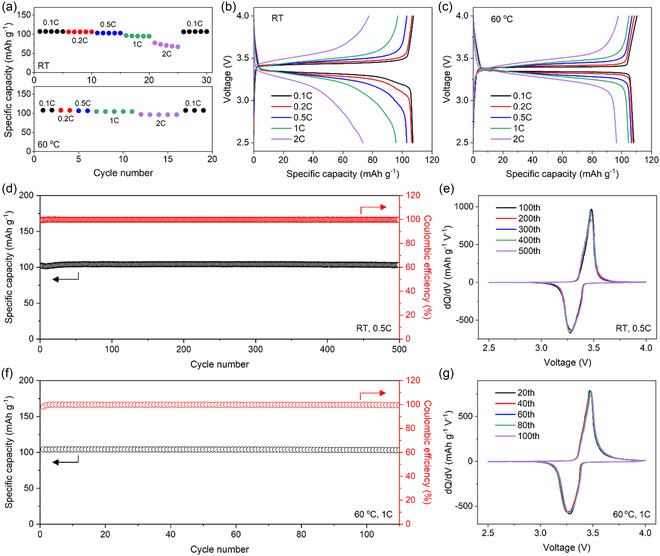
Electrochemical performance of Na_3_V_2_(PO_4_)_3_ (NVP)|Na cells using the TN5000 ionogel electrolyte. a) Specific discharge capacity at various charge–discharge rates at room temperature (top) and 60 °C (bottom). b,c) Voltage profiles at different rates at room temperature (b) and 60 °C (c). d) Specific discharge capacity and Coulombic efficiency for 500 cycles at 0.5 C at room temperature. e) Differential capacity (d*Q*/d*V*) curves for the 500 cycles at 0.5 C at room temperature. f) Specific discharge capacity and Coulombic efficiency for over 100 cycles at 1 C at 60 °C. g) d*Q*/d*V* curves for the 100 cycles at 1 C at 60 °C.

NVP|Na cells were assembled using the TN5000 ionogel electrolyte, and their rate and cycling performance were assessed. **Figure** **6**a displays their specific discharge capacity measured at various charge–discharge rates at room temperature and 60 °C. At room temperature, the specific discharge capacity at 0.1 C was 107 mAh g^−1^, corresponding to 91% of the theoretical capacity of NVP. More than 90% of the capacity at 0.1 C was maintained at 1 C, showing desirable rate capability. This performance surpasses that of previously reported NVP|Na cells employing ionogel electrolytes, in terms of current density and areal capacity (Figure S14, Supporting Information), considering specific capacity and active mass loading simultaneously.^[^ ^33^, ^34^, ^35^, ^36^ ^]^ The improved performance can be attributed to the favorable Na‐ion conductivity of the TN5000 ionogel electrolyte, which allows increased active mass loading without significant loss of specific capacity. At 60 °C, the cells delivered a similar capacity at 0.1 C but exhibited improved capacities at rates higher than 1 C. This improvement can be ascribed to the enhanced ionic conductivity at the elevated temperature (Figure S15, Supporting Information), as supported by the observation that, compared to the cells at room temperature (Figure 6b), those operated at 60 °C (Figure 6c) showed decreased overpotentials during the charging and discharging processes.

Figure 6d presents the cycling performance of NVP|Na cells using the TN5000 ionogel electrolyte for 500 cycles at 0.5 C at room temperature. The NVP|Na cells exhibited a capacity retention of over 99% and an average Coulombic efficiency of 99.8% for the 500 cycles, with minimal shifting of the charging and discharging voltages (Figure 6e and Figure S16, Supporting Information). Even at 60 °C, the cells retained more than 99% of their initial capacity over 100 cycles at 1 C (Figure 6f), with an average Coulombic efficiency of 99.8% and stable voltage profiles (Figure 6g and Figure S17, Supporting Information). This outstanding cycling stability can be related not only to suppressed dendrite growth on Na anodes but also to the electrochemical stability of the electrolytes. In particular, the ionogel electrolyte showed improved anodic stability (Figure S18, Supporting Information) compared to the IL alone since the effective immobilization of IL anions by talc nanosheets suppresses their oxidation at high‐potential electrode surfaces.^[^ ^37^, ^38^ ^]^ This enhanced electrochemical stability allowed the ionogel electrolyte to be used in constructing SMBs with a higher‐potential cathode material, Na_3_V_2_(PO_4_)_2_F_3_ (NVPF). When tested with a voltage window of 2.0–4.5 V, NVPF|Na cells using the IL alone (Figure S19, Supporting Information) exhibited severe degradation and incomplete charging due to the oxidation of the IL electrolyte beyond its anodic stability limit. In contrast, cells employing the TN5000 ionogel electrolyte (Figure S20, Supporting Information) were successfully operated with typical charge–discharge voltage profiles, confirming the enhanced electrochemical stability of the ionogel electrolyte.

## 3 Conclusion

We have formulated ionogel electrolytes using size‐controlled talc nanosheets and a Na‐based IL for SMBs. As their lateral dimensions and thickness decrease, solution‐exfoliated talc nanosheets increase their surface area, enabling more effective immobilization of the IL and promoting the solidification of the ionogels, which leads to increased mechanical modulus. The smaller nanosheet size and enhanced modulus contribute to the distribution of nucleation sites for Na deposition and strengthen the mechanical resistance of the ionogel electrolytes against vertical Na dendrite growth. While exhibiting high mechanical strength, the ionogels maintain Na‐ion conductivity comparable to that of the neat IL since smaller talc nanosheets enhance the Na‐ion transference number. Consequently, the ionogel electrolytes based on the size‐controlled talc nanosheets facilitate the fabrication of NVP|Na cells with favorable rate capability at room temperature and excellent cycling stability, achieving over 99% capacity retention for 500 cycles. This study highlights the potential of size‐controlled talc nanosheets as solid matrices for designing ionogel electrolytes for high‐performance solid‐state SMBs. Moreover, although demonstrated here with talc, the sodium dendrite growth and suppression mechanisms is likely applicable to a broad range of nanoscale solid matrices for ionogel electrolytes.

## 4 Experimental Section

4.1

4.1.1

##### Exfoliation of Talc Nanosheets

A dispersion was prepared by mixing 50 g of bulk talc microparticles (Aladdin), 5 g of ethyl cellulose (4 cP viscosity grade, Sigma‐Aldrich), and 400 mL of ethanol, followed by sonication at 40 Hz with an ultrasonic power density of 0.5 W cm^−2^ at 10 °C for 12 h. The sonicated mixture was centrifuged (J‐26S XP, Beckman Coulter) at 200, 300, or 5000 rpm for 20 min to remove large talc particles, and the supernatant was collected. To induce flocculation of talc nanosheets and ethyl cellulose, the supernatant was mixed with a 40 mg mL^−1^ sodium chloride aqueous solution (2:1 by volume). After centrifugation at 7500 rpm for 10 min, the sedimented talc nanosheets with ethyl cellulose were rinsed with deionized water to eliminate residual sodium chloride, dried in a convection oven at 80 °C, and ground with a mortar and pestle to obtain a dry powder. The talc nanosheet/ethyl cellulose powder was annealed in a box furnace (BF51894C‐1, Thermo Scientific) at 400 °C for 4 h to decompose ethyl cellulose. The talc nanosheets were observed using SEM (Gemini 450, Zeiss). Their elemental composition and crystal structure were analyzed using an X‐ray photoelectrometer (VersaProbe III, PHI) and an X‐ray diffractometer (MiniFlex, Rigaku), respectively. The lateral size and thickness of the talc nanosheets were characterized using an AFM (Dimension Icon, Bruker), and their specific surface area was measured by Brunauer–Emmett–Teller (BET) analysis using an adsorption analyzer (ASAP 2020 PLUS HD88, Micromeritics).

##### Formulation and Characterization of Ionogels

To prepare a Na‐based IL, 1 M NaTFSI salt (99.95% purity, trace metal basis) was dissolved in Py13FSI (H_2_O ≤ 20 ppm, Solvionic). The talc nanosheets and IL were mixed using a mortar and pestle to produce ionogels. The solid matrix‐to‐IL weight ratio was set to 2:3 to balance mechanical modulus and ionic conductivity. The ionogels were used after aging for longer than 12 h. All of the ionogel preparation steps were carried out in an argon‐filled glovebox. The viscoelastic properties of the ionogels were measured using a rheometer (MCR 302e, Anton Paar) equipped with an 8 mm‐diameter parallel plate (gap between the rheometer stage and parallel plate: 1 mm) with a strain of 0.1% at 25 °C. To assess the ionic conductivity, the ionogels were sandwiched between two stainless steel disks, and their bulk resistance (*R*) was measured using a potentiostat (VSP, BioLogic) with a frequency range of 1 MHz−100 mHz and an amplitude of 10 mV. The ionic conductivity was calculated based on the following equation

$$\sigma =\frac{L}{R\times A}$$

where *L* and *A* are the thickness and area, respectively, of the ionogel electrolyte between the stainless steel electrodes. To evaluate the Na‐ion transference number (*t*_Na_), Na symmetric cells were fabricated and polarized at 10 mV, and their Nyquist plots were acquired before and after polarization. *t*_Na_ was calculated based on the following equation

$$t_{\text{Na}}=\frac{I_{S}\left(\Delta V-I_{0}R_{0}\right)}{I_{0}\left(\Delta V-I_{S}R_{S}\right)}$$

where Δ*V*, *I*, and *R* are the applied polarization voltage, current from polarization curves, and charge‐transfer resistance from the Nyquist plots. The subscripts 0 and S indicate the initial and steady states, respectively. The ionic conductivity and Na‐ion transference number at elevated temperatures were measured using an environment chamber (PM‐36, Lab Companion). The electrochemical stability was examined with a Na|electrolyte|stainless steel geometry by linear sweep voltammetry at a scan rate of 1 mV s^−1^ at room temperature. Na plating/stripping tests were executed using a Na|electrolyte|Cu geometry in CR2032‐type coin cells. After plating/stripping cycles, the coin cells were disassembled in the argon‐filled glovebox, and the Cu electrodes were rinsed with dimethyl carbonate and dried. The deposited Na was analyzed using FIB‐SEM (Helios 5 UC, Thermo Fisher Scientific) and EDS (Gemini 450, Zeiss) systems.

##### Battery Testing

To prepare NVP electrodes, NVP, carbon black, and poly(vinylidene fluoride) were purchased from MTI Corporation and mixed in a weight ratio of 8:1:1 using 1‐methyl‐2‐pyrrolidinone as a solvent. The slurry was blade coated onto aluminum foils and dried in a vacuum oven at 80 °C for over 12 h. The electrodes were cut into circles with a diameter of 10 mm for battery assembly, and the active mass loading was ≈4.0 mg cm^−2^. NVPF electrodes were prepared using the same procedure, with NVPF purchased from Hubei Sanxia Xingan Tech and an active mass loading of ≈3.5 mg cm^−2^. In the argon‐filled glovebox, the ionogel electrolyte was applied to Na metal anodes using a spatula, and the prepared cathode electrodes were placed onto the deposited ionogel electrolyte to fabricate CR2032‐type coin cells. Prior to assembly, the NVP and NVPF electrodes were submerged in the Na‐based IL for 5 min to improve interfacial contact with the ionogel electrolyte. All battery cells were measured using a battery testing system (LBT‐20 084, Arbin), with 1C defined as 117 and 128 mAh g^−1^ for NVP and NVPF, respectively.

## Supporting Information

Supporting Information is available from the Wiley Online Library or from the author.

## Conflict of Interest

The authors declare no conflict of interest.

## Supporting information

Supplementary Material

## Data Availability

The data that support the findings of this study are available from the corresponding author upon reasonable request.
