# Supplementary material for: Size‐Controlled Talc Nanosheet Ionogel Electrolytes for Dendrite Suppression in Solid‐State Sodium Metal Batteries
Source: Small Sci. 2025 Aug 31;5(11):2500399. doi: 10.1002/smsc.202500399 (PMC12622431; doi:10.1002/smsc.202500399)
Supplement: Supplementary file 1 — Supplementary Material [file SMSC-5-2500399-s001.pdf]

Supporting Information

Size-Controlled Talc Nanosheet Ionogel Electrolytes for Dendrite Suppression in Solid-State Sodium Metal Batteries

*Yuxing Gu, Yair Ein-Eli, and Woo Jin Hyun**

Y. Gu, W. J. Hyun
Department of Materials Science and Engineering
Guangdong Technion − Israel Institute of Technology
Shantou, Guangdong 515063, China
E-mail: woojin.hyun@gtiit.edu.cn

Y. Gu, Y. Ein-Eli, W. J. Hyun
Department of Materials Science and Engineering
Technion – Israel Institute of Technology
Haifa 3200003, Israel

Y. Ein-Eli
Grand Technion Energy Program (GTEP)
Technion – Israel Institute of Technology
Haifa 3200003, Israel

Y. Ein-Eli
Israel National Institute of Energy Storage (INIES)
Technion – Israel Institute of Technology
Haifa 3200003, Israel

W. J. Hyun
Guangdong Provincial Key Laboratory of Materials and Technology for Energy Conversion
Guangdong Technion − Israel Institute of Technology
Shantou, Guangdong 515063, China

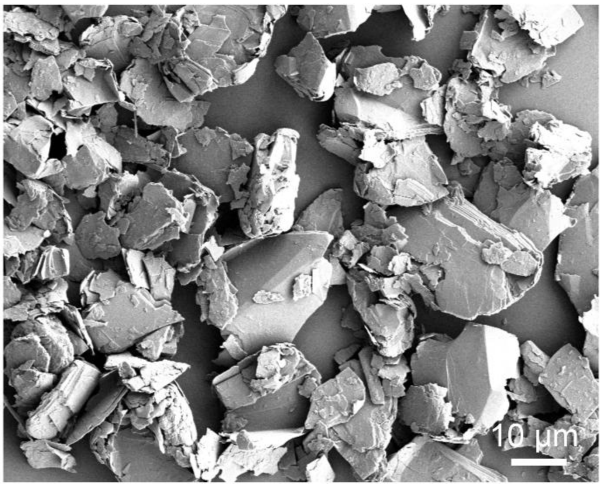

Figure S1. Scanning electron microscopy (SEM) image of bulk talc microparticles used for solution-based exfoliation of talc nanosheets.

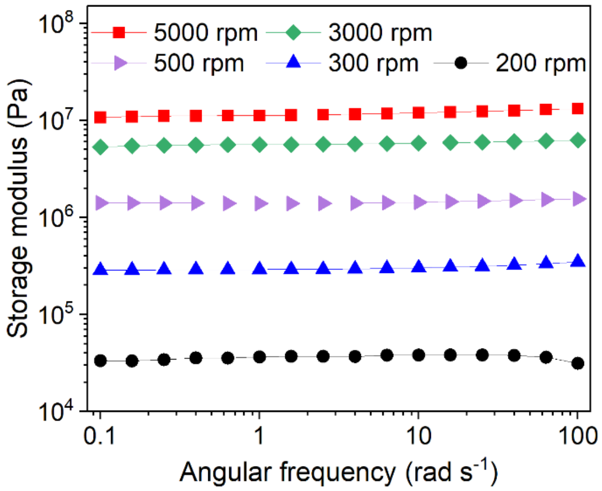

Figure S2. Storage modulus of ionogels prepared with talc nanosheets collected at centrifugation speeds ranging from 200 to 5000 rpm. Ionogels prepared with nanosheets collected at 200 rpm exhibited the lowest modulus, while those prepared at 5000 rpm showed the highest. Nanosheets collected at 300 rpm yielded an intermediate modulus. Hence, centrifugation speeds of 200, 300, and 5000 rpm were selected to represent ionogels with low, medium, and high mechanical moduli for investigating the influence of modulus on dendrite growth behavior.

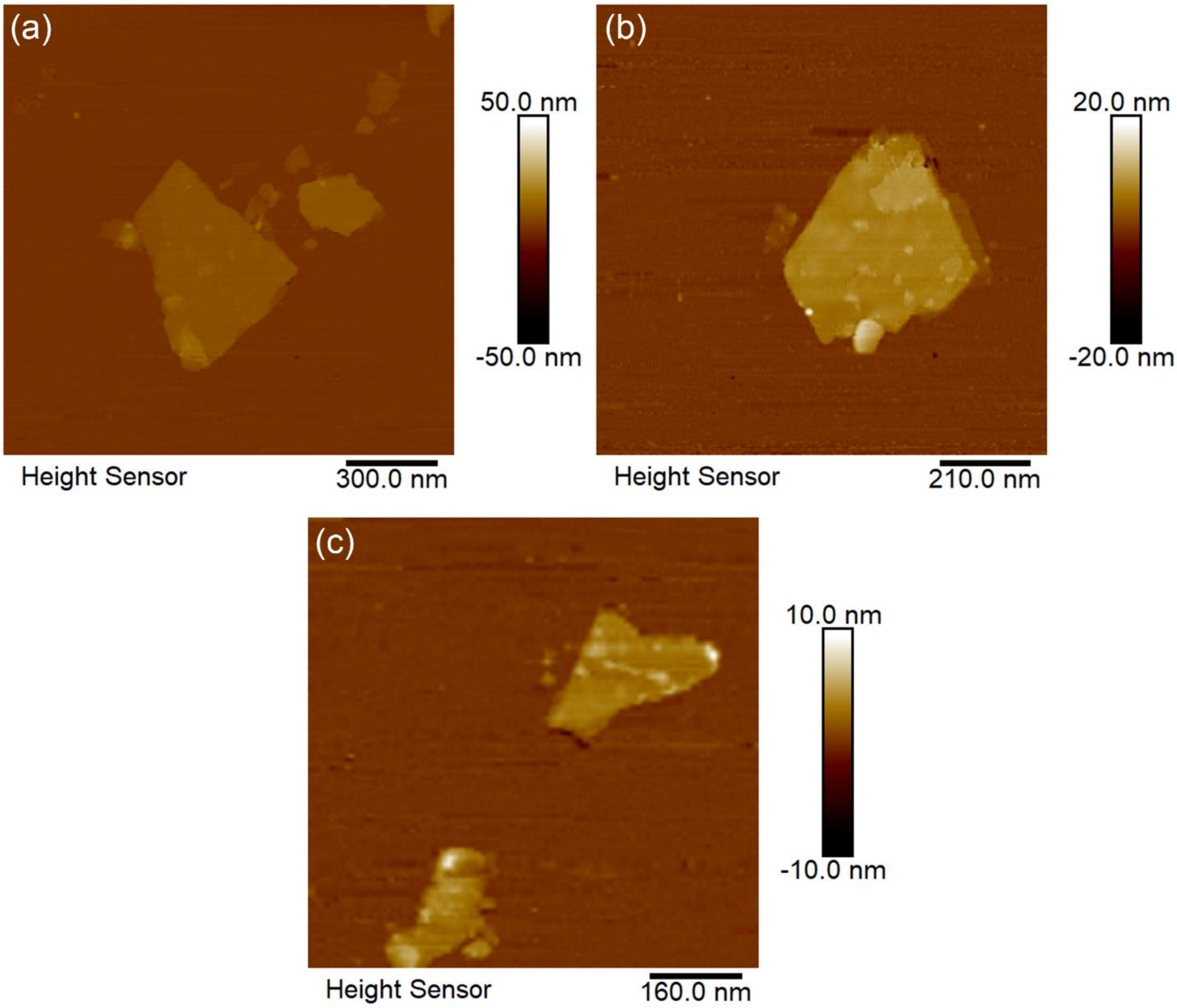

Figure S3. Representative atomic force microscopy (AFM) images of talc nanosheets exfoliated via a solution-based method and collected by centrifugation at different rotational speeds of 200 (a), 300 (b), and 5000 (c) rpm, referred to as TN200, TN300, and TN5000, respectively.

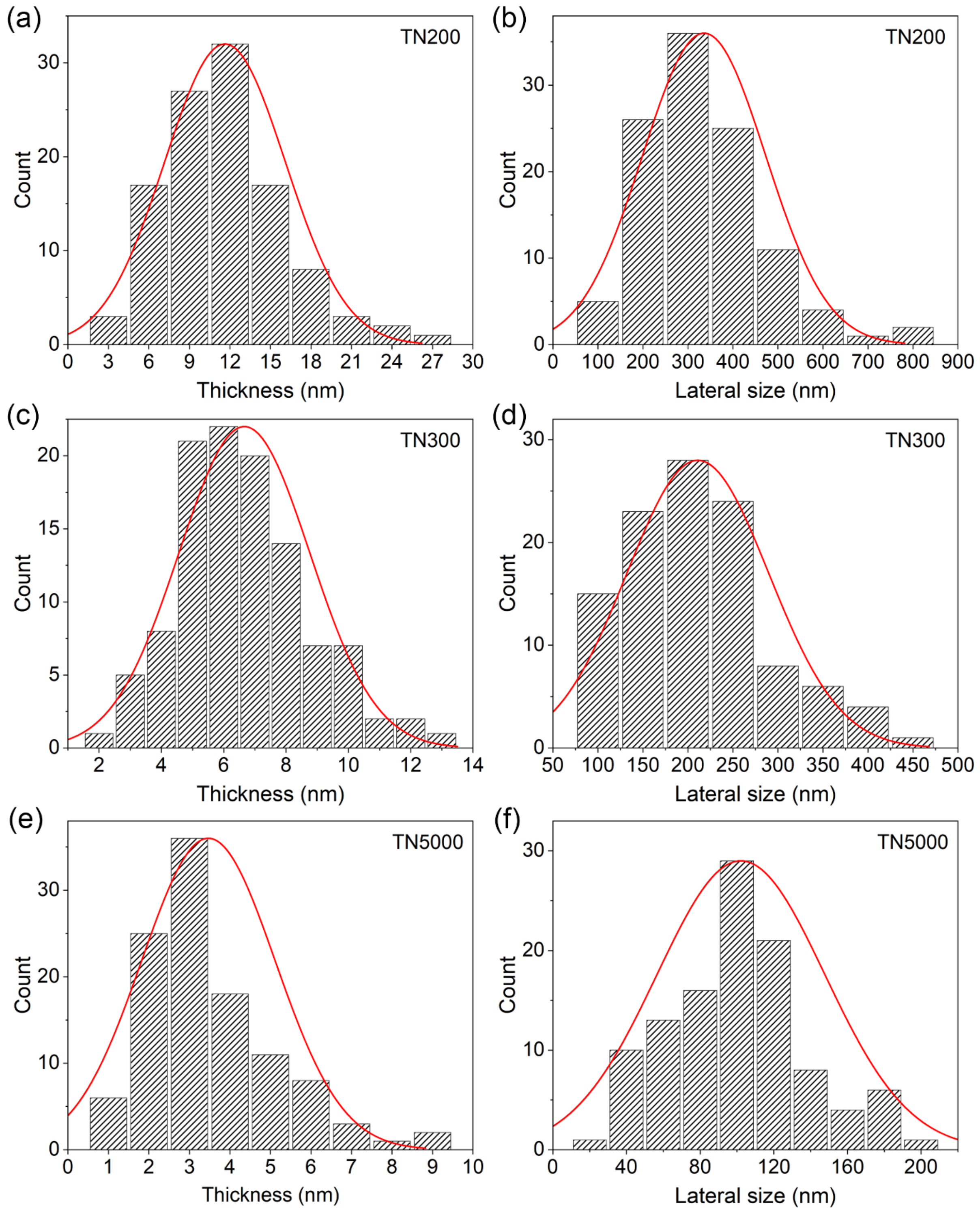

Figure S4. Thickness and lateral size distributions of 110 nanosheets for TN200 (a and b, respectively), TN300 (c and d, respectively), and TN5000 (e and f, respectively).

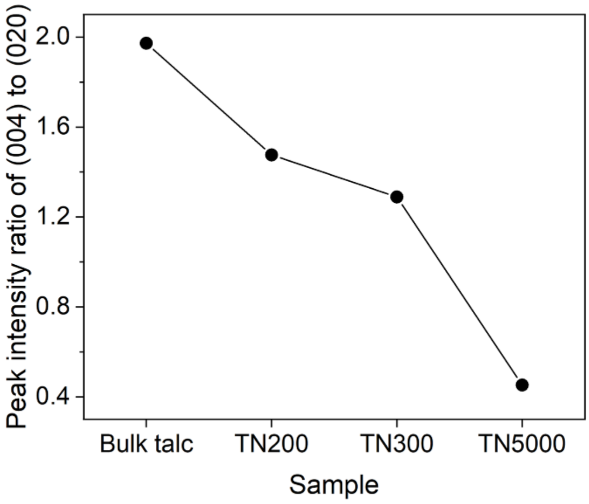

Figure S5. Relative peak intensity ratio of (004) to (020) in the X-ray diffraction (XRD) patterns of bulk talc, TN200, TN300, and TN5000.

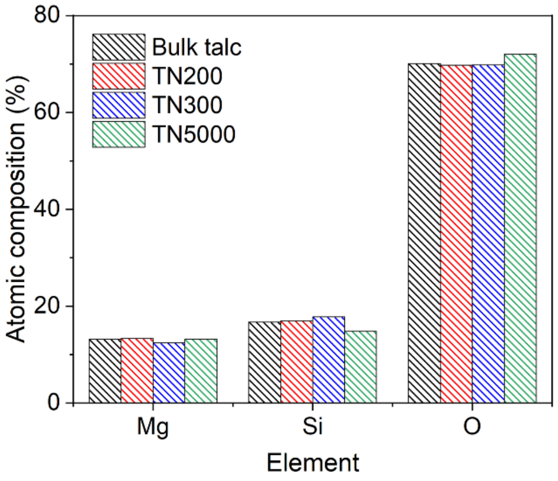

Figure S6. Atomic compositions of magnesium (Mg), silicon (Si), and oxygen (O) in bulk talc, TN200, TN300, and TN5000 characterized by X-ray photoelectron spectroscopy (XPS).

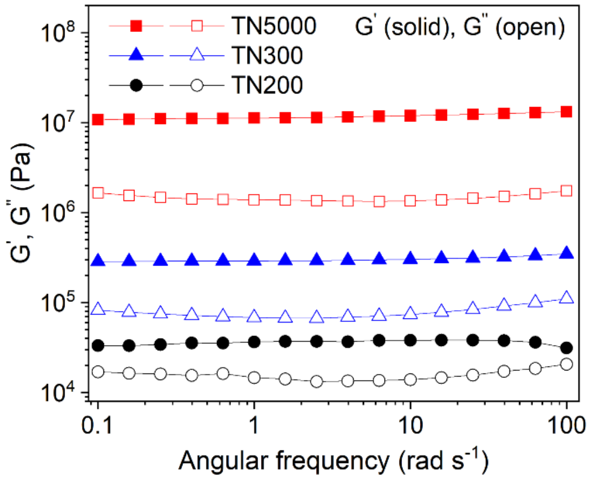

Figure S7. Storage (*G'*) and loss (*G''*) moduli of ionogels employing TN200, TN300, and TN5000 as solid matrices. The solid and open symbols represent *G'* and *G''*, respectively.

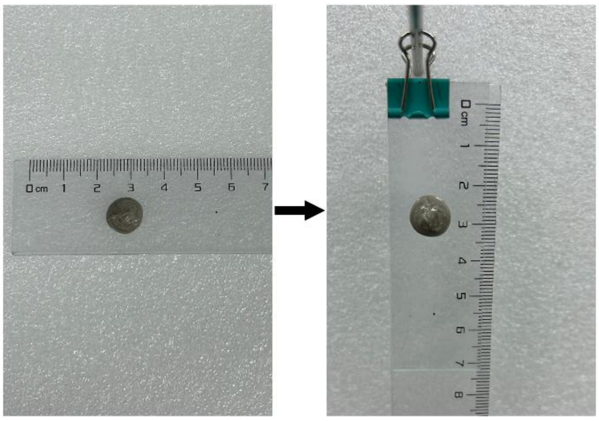

Figure S8. Photographs of a TN200 ionogel placed on a substrate, showing its solid-like behavior. After the substrate was tilted from a horizontal (left) to a vertical position (right), the ionogel remained adhered without flowing, indicating stable solid-like characteristics.

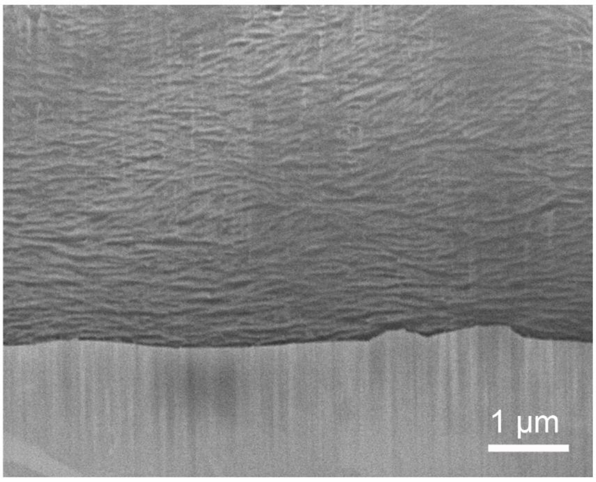

Figure S9. Cross-sectional SEM image of a TN5000 ionogel deposited on a Cu electrode.

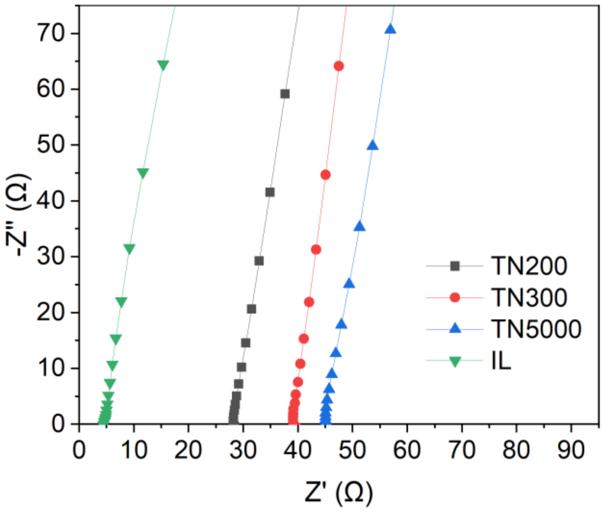

Figure S10. Electrochemical impedance spectra of stainless-steel|electrolyte|stainless-steel cells using the TN200, TN300, and TN5000 ionogel electrolytes, as well as the IL electrolyte, for ionic conductivity measurements.

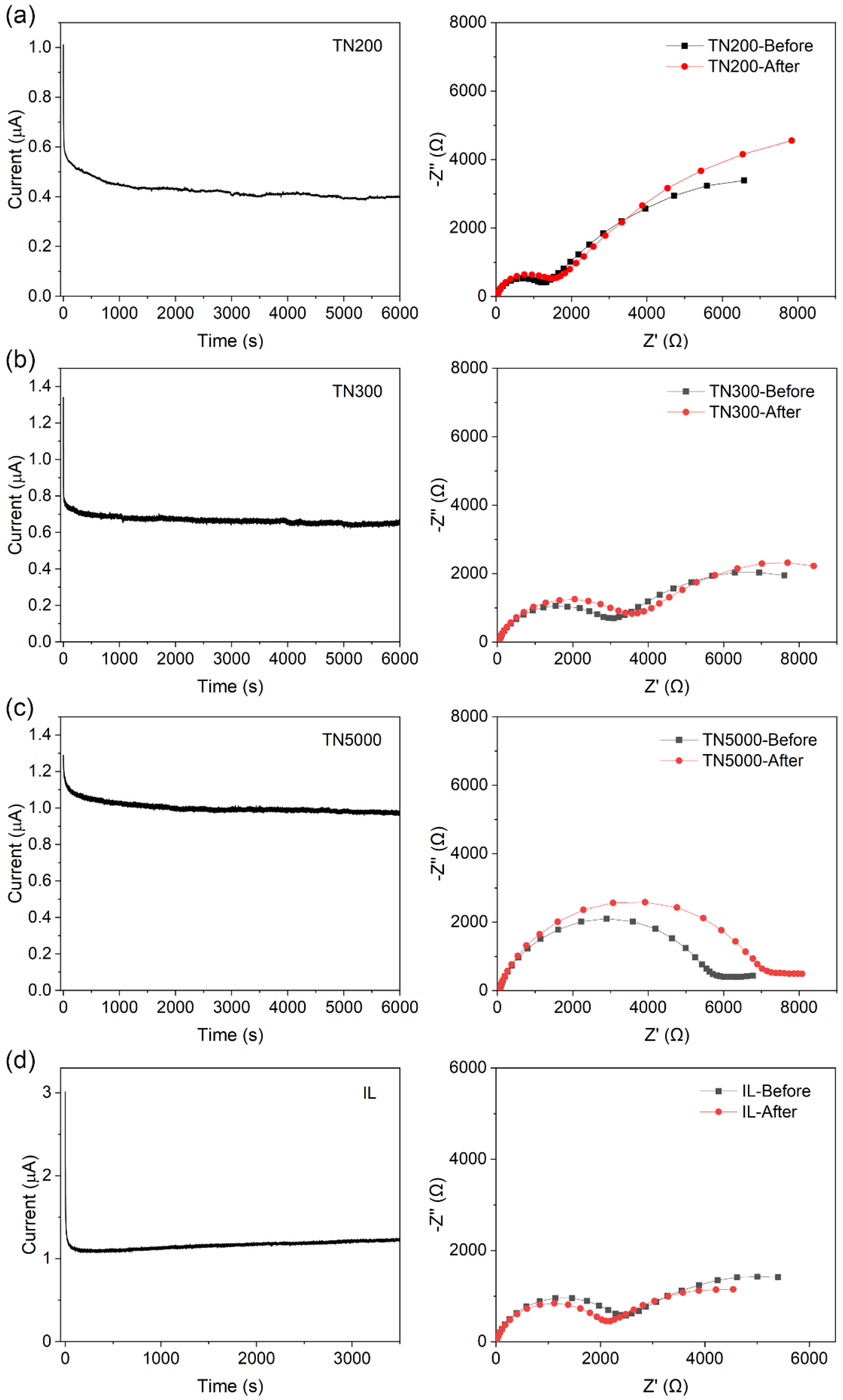

Figure S11. Polarization curves (left) and Nyquist plots (right) before and after polarization for Na sysmmetric cells using the TN200 (a), TN300 (b), TN5000 (c) ionogel electrolytes, as well as the IL electrolyte (d).

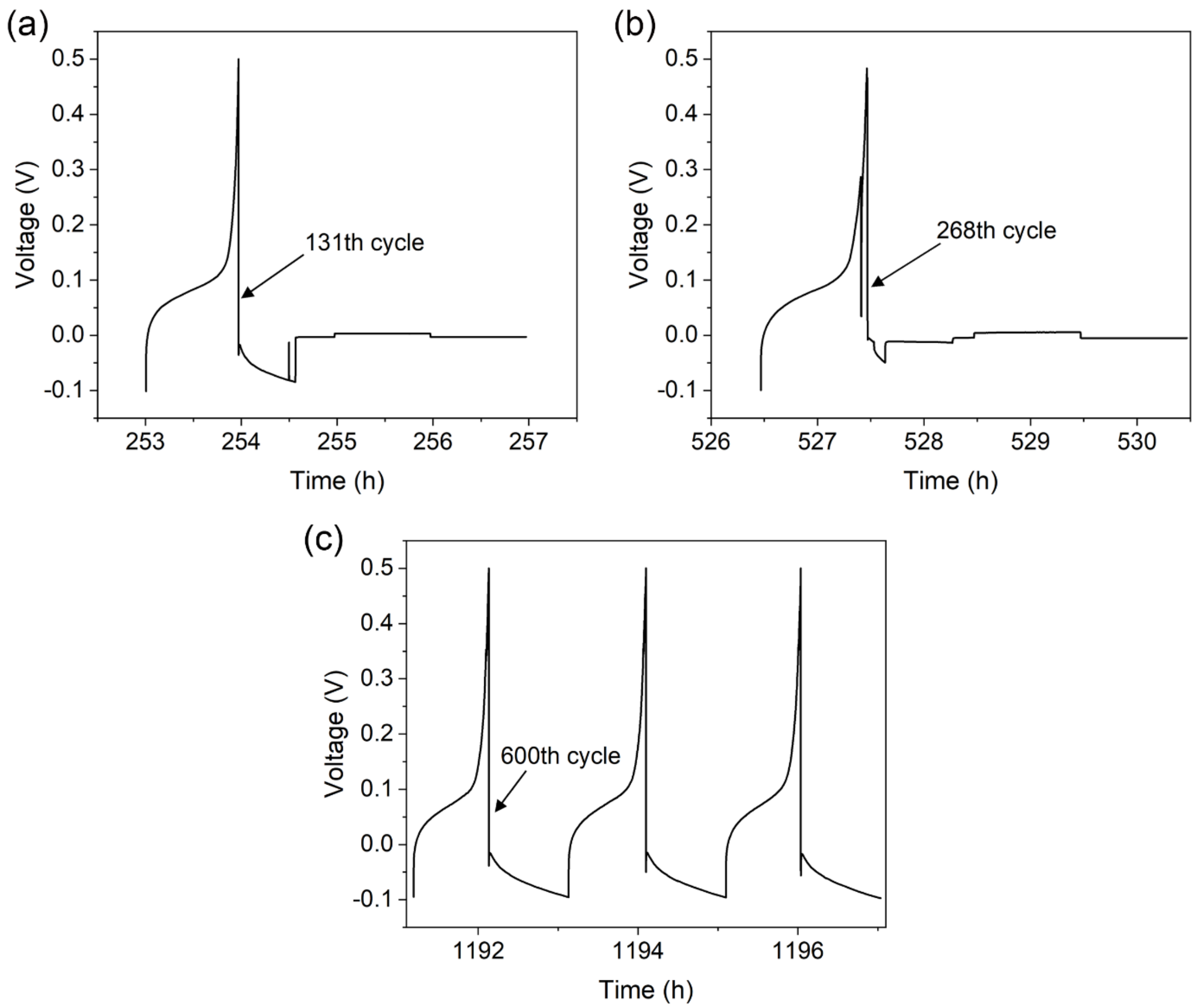

Figure S12. a,b) Voltage profiles of Na|Cu cells using the TN200 (a) and TN300 (b) ionogel electrolytes during the last cycles before short-circuit failure. c) Voltage profile of a Na|Cu cell employing the TN5000 ionogel electrolyte after 600 cycles.

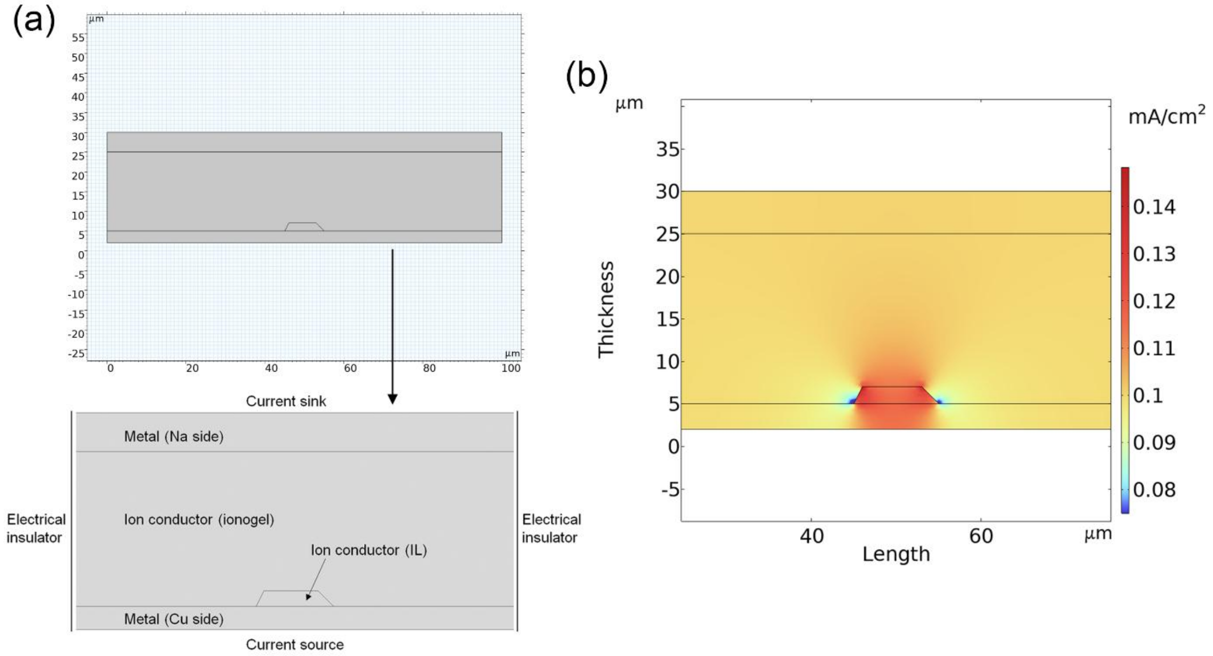

Figure S13. a) Geometric model used for the simulation of current density distribution in an ionogel with a confined IL region. The finite element simulation was performed using the electric current (EC) module in COMSOL Multiphysics. The two-dimensional geometry consists of a 20 μm-thick ion conductor (ionogel) slab sandwiched between a 5 μm-thick Na electrode and a 3 μm-thick Cu electrode. The quadrilateral in the ion conductor represents an IL region confined by talc nanosheets and the Cu electrode due to their irregular contact. The external surface of the Cu electrode is connected to a current source, and the external surface of the Na electrode is connected to a current sink. All other surfaces are defined as electrically insulating boundaries. To reflect the relatively higher conductivity of the IL compared to the bulk ionogel, the IL region and the remaining region of the ion conductor were approximated with conductivities of 1 and 0.5 mS cm^{-1}, respectively. b) Simulation result showing a localized increase in current density within the confined IL region in the ionogel. The simulation was performed with an applied current density of 0.1 mA cm^{-2}.

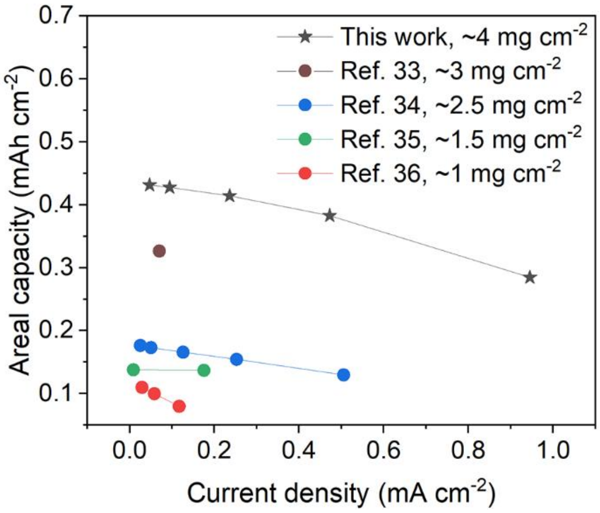

Figure S14. Areal capacity of the $Na_3V_2(PO_4)_3$ (NVP)|Na cell with the TN5000 ionogel electrolyte as a function of current density, in comparison to that of previously reported NVP|Na batteries using ionogel electrolytes. The areal mass values indicate the active material loadings used in the corresponding cells.

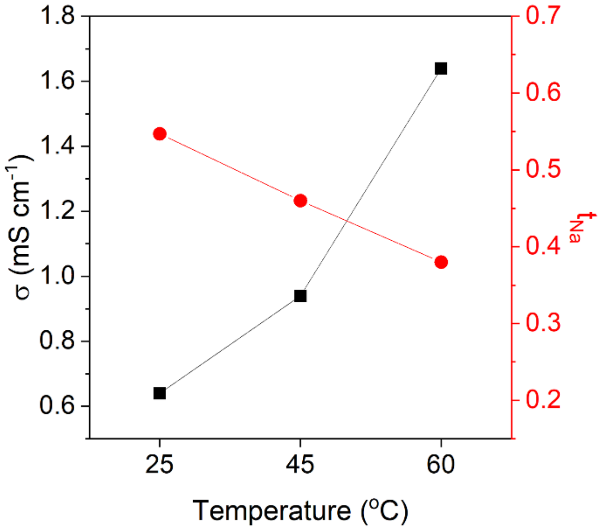

Figure S15. Ionic conductivity and Na-ion transference number of the TN5000 ionogel electrolyte at elevated temperatures.

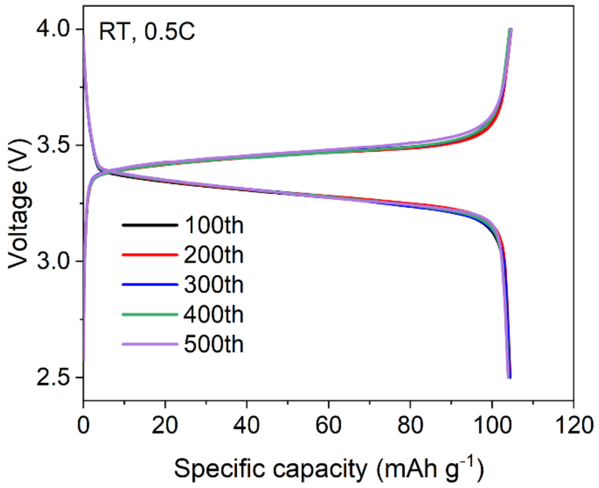

Figure S16. Voltage profiles of an NVP|Na cell using the TN5000 ionogel electrolyte for 500 cycles at 0.5C at room temperature.

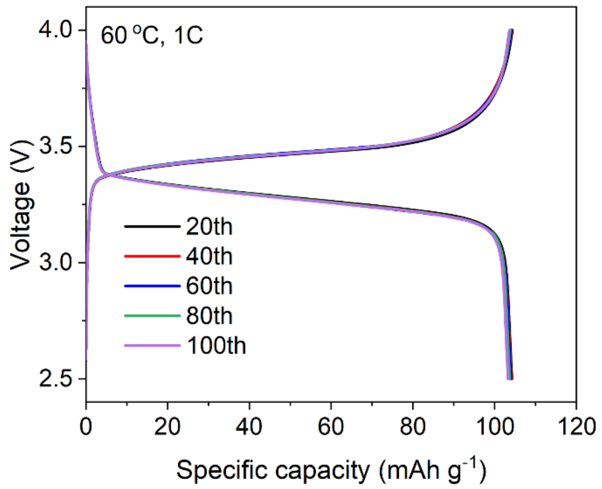

Figure S17. Voltage profiles of an NVP|Na cell employing the TN5000 ionogel electrolyte for 100 cycles at 1C at 60 °C.

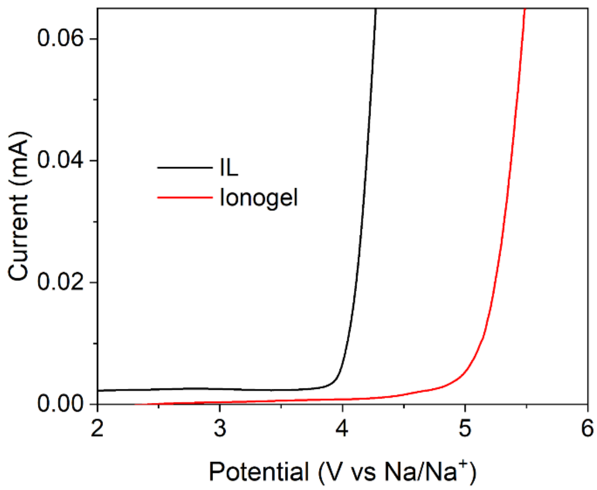

Figure S18. Linear sweep voltammetry curves of Na|stainless-steel cells using the IL and TN5000 ionogel electrolytes. As the voltage was increased from their open circuit voltage, the IL cell showed a significant current increase at ~4 V (vs Na/Na^+), originating from the oxidation of FSI anions. However, the ionogel cell exhibited significantly reduced oxidation current up to >4.5 V (vs Na/Na^+), indicating suppressed electrolyte decomposition and improved electrochemical stability of the ionogel electrolyte. The lower initial current observed in the ionogel cell is likely due to reduced ion mobility caused by interactions between the IL and the talc nanosheets.

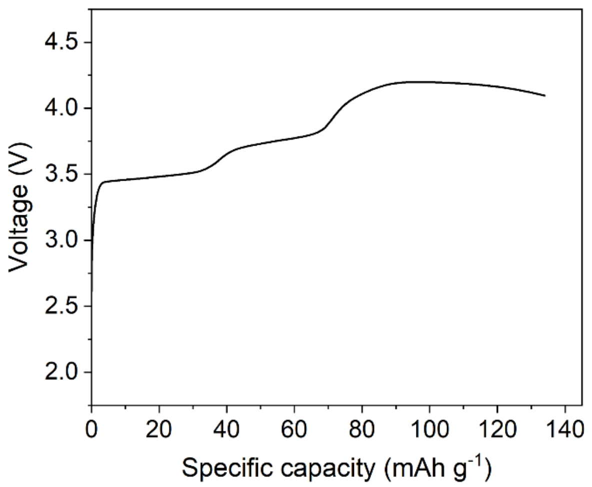

Figure S19. 1st cycle charge voltage profile of a $Na_3V_2(PO_4)_2F_3$ (NVPF)|Na cell using the IL electrolyte.

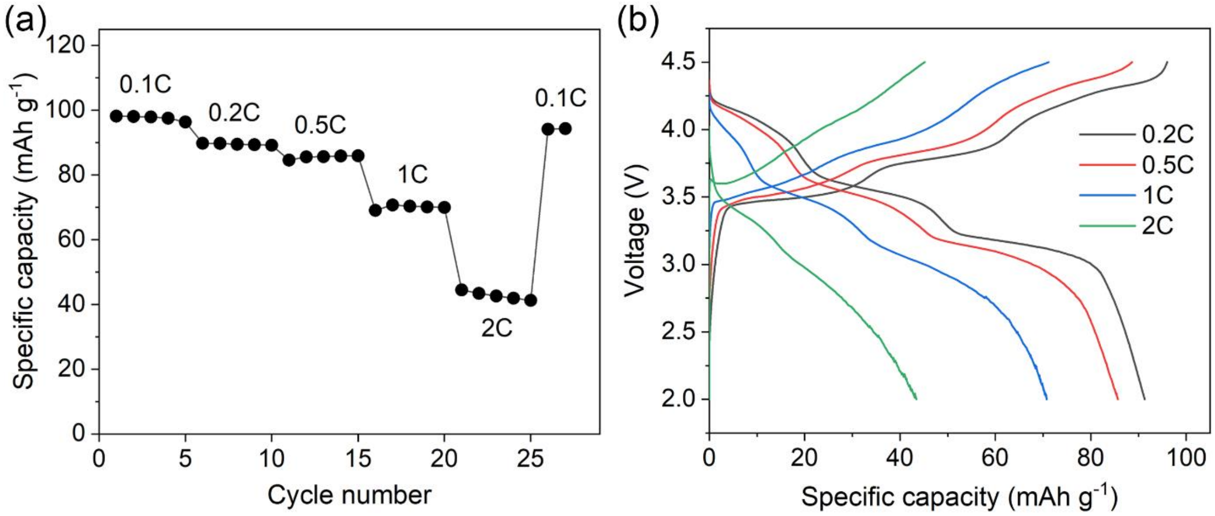

Figure S20. Specific discharge capacity (a) and voltage profiles (b) of an NVPF|Na cell employing the TN5000 ionogel electrolyte at various charge–discharge rates at room temperature.
